# Association between life’s essential 8 and diabetic kidney disease: a population-based study

**DOI:** 10.1080/0886022X.2025.2454286

**Published:** 2025-03-10

**Authors:** Cong Liu, Jiju Yang, Hongdian Li, Yuanyuan Deng, Shaoning Dong, Pengfei He, Jiao Zhang, Mianzhi Zhang

**Affiliations:** ^a^ Department of Nephrology, Dongfang Hospital of Beijing University of Chinese Medicine, Beijing, China; ^b^ Graduate School, Beijing University of Chinese Medicine, Beijing, China; ^c^ Department of Nephrology, Tianjin Academy of Traditional Chinese Medicine Affiliated Hospital, Tianjin, China; ^d^ Tianjin Famous Chinese Medicine Inheritance Workshop of Mianzhi Zhang, Tianjin, China

**Keywords:** Life’s essential 8, diabetic kidney disease, NHANES, cardiovascular health

## Abstract

**Background and Aims:**

Diabetic patients are highly susceptible to cardiovascular and renal diseases. As a newly updated comprehensive index for assessing cardiovascular health (CVH), Life’s essential 8 (LE8) has the potential to serve as a practical tool for evaluating the risk of diabetic kidney disease (DKD). We are committed to exploring the relationship between LE8 and its subscales with DKD in diabetic patients, aiming to provide preliminary evidence for the formulation of clinical strategies.

**Methods and Results:**

A total of 3,715 NHANES participants were included in this study, representing 18.9 million non-institutionalized residents of the United States. The mean age of all subjects was 59.72 years, and the weighted prevalence of DKD among diabetic patients was 36.39%. After adjusting for potential confounding factors, it was found that compared to the low LE8 group, the risk of developing DKD was significantly lower in the moderate LE8 group (OR: 0.54, 95% CI: 0.43-0.66) and the high LE8 group (OR: 0.18, 95% CI: 0.08-0.42). A similar trend was observed across the subscales of the LE8 score. The results of the fully adjusted restricted cubic spline regression analysis revealed a linear relationship between LE8 and its subscales with DKD. The findings remained consistent in subgroup and sensitivity analyses, with no significant interactions observed between subgroups.

**Conclusion:**

Higher scores on the LE8 and its subscales were associated with a lower risk of developing DKD. However, the long-term causal relationship between LE8 and DKD risk necessitates further validation and exploration through large-scale, rigorously designed prospective studies.

## 1. Introduction

Diabetic kidney disease (DKD) is one of the chronic microvascular complications caused by diabetes, and it has now become a significant cause of chronic kidney disease (CKD) and end-stage renal disease [1]. It severely threatens patient health and reduces the quality of life. It is predicted that by 2040, there will be approximately 642 million diabetic patients worldwide [2], and more than 35% of them will develop kidney damage [3]. DKD increases the cost of healthcare and poses a substantial burden on global public health [4]. Studies have shown that the risk of death in DKD patients is particularly closely associated with cardiovascular disease (CVD) [5]. Increased urinary albumin excretion and a decreased estimated glomerular filtration rate (eGFR) both increase the risk of CVD and death in DKD patients [6]. For patients with an eGFR of less than 60 mL/min/1.73 m^2^, CVD is the most common cause of death, particularly heart failure and valve disease [7].

Although numerous studies have shown that hyperglycemia, hypertension, dyslipidemia, and smoking are common pathogenic factors for both CVD and DKD [8, 9], these traditional single risk factors do not fully explain the complexity of CVD’s impact on DKD. In 2010, the American Heart Association (AHA) introduced the concept of Life’s simple 7 (LS7), which has since become a powerful tool for assessing cardiovascular health (CVH) [10]. However, the original definitions and quantitative standards of the components of LS7 are less sensitive than ideal to interindividual differences and intraindividual variations. In 2022, the AHA updated LS7 to Life’s essential 8 (LE8) [11], which includes four behavioral elements—diet, physical activity, tobacco/nicotine exposure, and sleep, as well as four health factors—Body Mass Index (BMI), lipids, glucose, and blood pressure. The calculation method has been updated to better reflect interindividual and intraindividual differences. Epidemiological studies have shown that as LE8 scores increase, the risk of CVD decreases accordingly [12–14].

Given the close relationship between DKD and CVD, promoting CVH may be a crucial pathway for the risk management of DKD. The National Health and Nutrition Examination Survey (NHANES) database is the primary data source recommended by the AHA for tracking CVH in the United States population, yet no studies to date have been conducted using NHANES to analyze the relationship between LE8 and DKD. Therefore, this study utilizes NHANES for a cross-sectional analysis aimed at exploring the correlation between LE8 and DKD, with the goal of providing a more comprehensive strategy and evidence base for the prevention and management of DKD.

## 2. Methods

This study was conducted in accordance with the Strengthening the Reporting of Observational Studies in Epidemiology (STROBE) statement [15].

### 2.1. Data source and study participants

The data for this study were sourced from the NHANES database (https://wwwn.cdc.gov/nchs/nhanes/Default.aspx). NHANES represents a large cross-sectional survey of the United States population, conducted using a complex, stratified, multistage sampling approach. Investigators collected a comprehensive array of information from participants through home interviews, laboratory testing, and other methods (detailed methods can be found in the NHANES survey manuals and analysis guidelines). This dataset provides a representative reflection of the health and nutritional status of the noninstitutionalized civilian population. NHANES has received approval from the Ethics Review Board of the National Center for Health Statistics, and all participants provided written informed consent.

**Figure 1.**
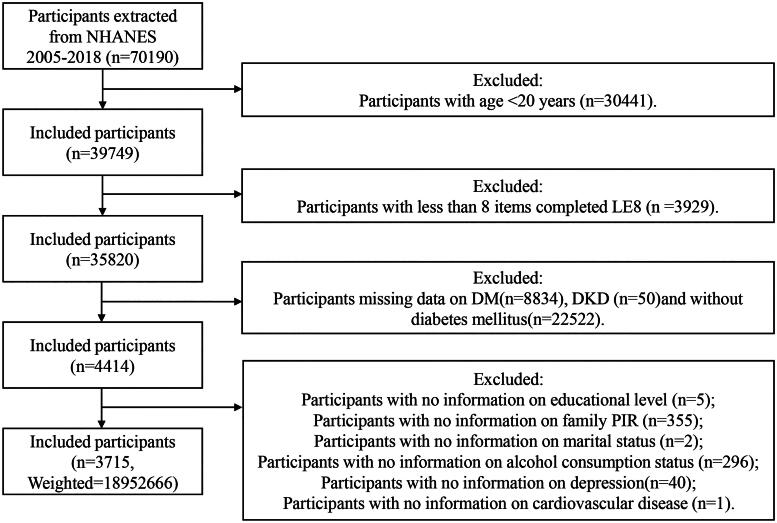
Participant selection flowchart.

Utilizing the publicly available data from NHANES, in conjunction with the objectives of the study, we analyzed data from seven survey cycles spanning the period of 2005-2018. Participants were selected based on the following criteria: individuals were excluded (1) < 20 years; (2) unavailable data on LE8; (3) unavailable data on diabetes mellitus (DM) or without DM; (4) unavailable data on DKD; (5) pregnant; (6) incomplete data of covariates. The screening process is depicted in Figure 1.

### 2.2. Definition of life’s essential 8

The LE8, developed by the AHA based on the LS7, encompasses four health behaviors—diet, physical activity, tobacco/nicotine exposure, and sleep, as well as four health factors: BMI, non-high-density lipoprotein cholesterol, blood glucose, and blood pressure [11]. Assessment of dietary habits was conducted utilizing the Healthy Eating Index 2015, with data acquired *via* a 24-h dietary recall and quantified using the United States Department of Agriculture Automated Multiple-Pass Method (refer to Supplementary Table 1). The administered questionnaire collected information on levels of physical activity (self-reported minutes of moderate or vigorous physical activity per week), tobacco/nicotine exposure (self-reported use of cigarettes or inhaled nicotine-delivery system), sleep patterns (self-reported average hours of sleep per night), and the status of diabetes. Data regarding blood lipids and glucose levels were extracted from laboratory reports. Measurements for blood pressure, height, and weight were obtained at the Mobile Examination Center. The usage of medications for treating hypercholesterolemia, hypertension, and diabetes was also self-reported. BMI was calculated by dividing an individual’s weight in kilograms by the square of their height in meters.

The LE8, each with a score ranging from 0 to 100 points, are obtained by calculating the unweighted average of the 8 subitems (refer to Supplementary Table 2). A total score below 50 indicates ‘poor’ CVH, scores ranging from 50 to 79 are considered ‘moderate’, and scores of 80 or higher signify ‘high’ CVH. In this study, we employed the same definitions and cutoff points to measure and categorize the scores of health behaviors and health factors, in order to further investigate the relationship between the LE8 subscales and DKD.

### 2.3. Definition of diabetic kidney disease

The diagnosis of DM [16]: the use of diabetes medication or insulin, a diagnosis by a doctor, glycated hemoglobin HbA1c ≥ 6.5%, fasting glucose ≥ 7 mmol/L (126 mg/dL), 2-h oral glucose tolerance test with blood glucose ≥ 11.1 mmol/L (200 mg/dL). The DKD was defined as the diabetes combined with albuminuria (urinary albumin-to-creatinine ratio (ACR) ≥ 30 mg/g) and/or low-eGFR (eGFR < 60 mL/min/1.73 m^2^) according to the KDIGO 2021 Guidelines[17]. For eGFR calculation, we applied the CKD-Epidemiology Collaboration equation as follows [18]: GFR = 141·min [Scr/κ,1]α × max [Scr/κ, 1] − 1.209 × 0.993 Age × 1.018 [if women] × 1.159 [if black]); κ was 0.7(women) or 0.9(men), a was − 0.329 (women) or − 0.411(men), and min/max indicate the minimum/maximum of Scr/κ or 1.

### 2.4. Assessment of covariates

Mixture effect is an important issue in multivariate analysis, which is crucial for ensuring the stability and robustness of the results. Based on previous studies and considering the clinical significance, we have included some covariates that might affect the research outcomes. Demographic factors include age, sex, race (Non-Hispanic White, Non-Hispanic Black, Mexican American, and Other), educational level (less than high school, high school diploma, more than high school), marital status (divorced/separated/widowed, married/living with a partner, never married), and the family poverty income ratio (PIR) (< 1.3, 1.3 − 3.5, > 3.5). Medical history includes CVD (coronary heart disease, congestive heart failure, heart attack, stroke, angina), depression (assessed by Patient Health Questionnaire-9 scale). Alcohol consumption status (never, former, current) was also considered.

### 2.5. Statistical analysis

Due to the complex stratified sampling design employed by the NHANES, the data were weighted according to the NHANES-recommended sample weight calculation method (weight selection: dietary day one sample weight). The data from 2005 to 2018 were merged, with the 14-year weights equaling one-seventh of the 2-year weights. The means, standard deviations, percentages, and *p*-values reported in this study are all weighted values. The weighting method corrects for sample imbalances, making the model estimates more accurate and representative. A Shapiro-Wilk statistical test was used to confirm whether or not continuous variables have a normal distribution. In the baseline characteristics table, participants were divided into three groups based on the LE8 level. Continuous variables are presented as mean and standard deviation (variables with skewed distributions are represented by medians and quartiles), and categorical variables are presented as percentages. Chi-square tests and Analysis of Variance were used to test categorical and continuous variables, respectively. Age-standardized prevalence rates and standard errors were calculated for the total LE8 score, health behaviors score, and health factors score.

We employed weighted multivariate logistic regression analysis to explore the relationship between LE8 score, health behaviors score, and health factors score with DKD. Variance inflation factor (VIF) was used to assess collinearity between variables. The VIF measures the increase in variance of an estimated regression coefficient due to collinearity. A general rule of thumb is that VIF values > 5 indicate a problematic level of collinearity, which can lead to unstable regression results and weaken predictive capabilities [19]. LE8, health behaviors score, and health factors score were entered as continuous variables (per 10-point increase) and as categorical variables (low, moderate, high CVH) to calculate odds ratios (OR). Model 1 adjusted for gender, age, marital status, race, educational level, and family PIR; model 2 adjusted for model 1 and alcohol consumption status; model 3 adjusted for all covariates. Additionally, we used restricted cubic spline regression to observe potential dose-response relationships. To further investigate the relationship between LE8 and DKD in different populations, we conducted subgroup analyses by age, gender, glycohemoglobin level, CVD, hypertension, hyperlipidemia, depression and alcohol use, and performed interaction tests. We also conducted sensitivity analyses to verify the stability of our results. Specifically, we repeated the weighted multivariable logistic regression analysis after excluding patients with CVD and after addressing missing data using multiple imputation with 5 repetitions and chained equations. All statistical analyses were performed using R version 4.3.1. Statistical significance was defined as two-tailed *p* < 0.05.

## 3. Results

### 3.1. Baseline characteristics

Table 1 presents the baseline characteristics of 3,715 participants grouped by CVH, representing approximately 18.95 million the United States adults aged ≥ 20 years with diabetes. The mean age of all subjects was 59.72 years, with 50.45% males and 49.55% females. The weighted prevalence of DKD among diabetic patients was 36.39%. Compared to the low and moderate CVH groups, the high CVH group had a higher proportion of participants from other races, married/partnered individuals, and those who never drank/currently drink alcohol. Additionally, they were younger, had higher levels of education and wealth, lower levels of ACR, higher levels of eGFR, and lower prevalence of CVD, depression, and DKD (*p* < 0.05). No significant differences were observed in gender among the groups.

**Table 1.** Baseline characteristics of the study population.

Characteristics	Overall	Low	Moderate	High	*p*
Participant number (weighted)	n= 18952666	n= 6102587	n= 12274297	n= 575782	
Age, years	59.72 (13.35)	59.12 (12.28)	60.31 (13.71)	53.85 (14.82)	0.0025
Sex, %					0.1417
Male	50.45	47.02	52.27	48.19	
Female	49.55	52.98	47.73	51.81	
Race, %					<0.0001
Non-Hispanic White	66.61	66.99	66.69	61.01	
Non-Hispanic Black	13.35	17.13	11.73	7.62	
Mexican American	8.14	7.06	8.93	2.93	
Other	11.90	8.82	12.65	28.44	
Education level, %					<0.0001
Less than high school	20.68	27.75	17.77	7.89	
High school diploma	26.62	28.53	26.08	18.07	
More than high school	52.69	43.72	56.15	74.04	
Marital status, %					0.0205
Divorced/separated/widowed	27.00	30.55	25.62	19.03	
Married/living with a partner	65.19	59.95	67.48	71.79	
Never married	7.81	9.50	6.90	9.19	
Family PIR, %					<0.0001
<1.3	23.48	35.56	18.08	10.69	
1.3-3.5	39.71	38.62	40.84	27.16	
≥3.5	36.81	25.83	41.08	62.15	
Cardiovascular disease, %					<0.0001
No	74.99	66.88	78.36	88.89	
Yes	25.01	33.12	21.64	11.11	
Depression, %					<0.0001
No	89.08	82.43	92.07	95.90	
Yes	10.92	17.57	7.93	4.10	
Alcohol consumption, %					<0.0001
Never	13.98	12.39	14.46	20.62	
Former	23.33	30.94	20.34	6.55	
Current	62.69	56.698	65.19	72.82	
AHA LE8 scores					
Total LE8 score	55.67 (13.20)	40.60 (7.12)	61.89 (7.59)	82.62 (3.31)	<0.0001
Health behaviors score	63.23 (19.04)	45.06 (14.77)	71.05 (13.85)	89.00 (7.84)	<0.0001
Diet score	50 (25,80)	25 (0,50)	50 (25,80)	80 (50,80)	<0.0001
Physical activity score	80 (0,100)	0 (0,60)	100 (40,100)	100 (100,100)	<0.0001
Smoke score	75 (75,100)	75 (0,100)	100 (75,100)	100 (80,100)	<0.0001
Sleep score	80.85 (25.79)	70.23 (30.33)	85.46 (21.88)	95.02 (10.25)	<0.0001
Health factors score	48.11 (15.89)	36.14 (12.51)	52.74 (13.34)	76.22 (9.56)	<0.0001
BMI score	30 (15,70)	15 (0,30)	30 (30,70)	70 (70,100)	<0.0001
Blood lipids score	60 (40,80)	40 (20,80)	80 (40,80)	80 (80,100)	<0.0001
Blood glucose score	40 (30,40)	30 (20,40)	40 (30,60)	40 (40,100)	<0.0001
Blood pressure score	50 (30,80)	30 (25,55)	55 (30,80)	80 (75,100)	<0.0001
ACR, mg/g	11.06 (6.12, 31.45)	16.06 (7.67, 58.91)	9.60 (5.71, 23.71)	6.93 (4.03, 11.95)	<0.0001
eGFR, mL/min/1.73 m^2^	82.13 (24.25)	81.32 (25.74)	82.05 (23.50)	92.33 (21.46)	0.0017
DKD, %					<0.0001
No	63.61	53.24	67.56	89.15	
Yes	36.39	46.76	32.44	10.85	

PIR: poverty income ratio; AHA: American Heart Association; LE8: life’s essential 8; BMI: body mass index; ACR: urinary albumin-to-creatinine ratio; eGFR: estimated glomerular filtration rate; DKD: diabetic kidney disease.

### 3.2. Association of the LE8 and its subscales with diabetic kidney disease

As shown in Supplementary Table 3, all covariates exhibit VIF values less than 5, suggesting that collinearity has a minimal impact on the results.

**Figure 2.**
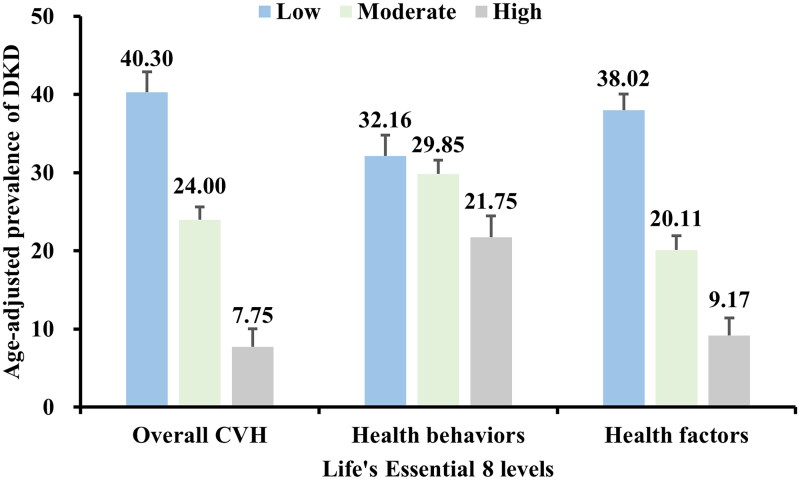
Age-adjusted prevalence of DKD in different levels of LE8 scores.

The age-adjusted prevalence of DKD (Figure 2) demonstrated that participants with higher scores, whether in overall LE8 or its subscales of health behaviors scores and health factors scores, had a lower prevalence of DKD compared to those with moderate and low scores.

**Figure 3.**
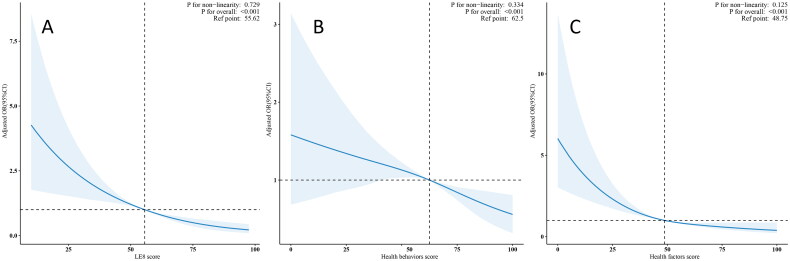
The linear associations between LE8 and its subscales with DKD.

We further examined the relationship between LE8 and its subscales scores with DKD using a weighted multivariable logistic regression model (Table 2). In the fully adjusted model, the results indicated a significantly reduced risk of DKD in the moderate LE8 group (OR:0.54, 95% CI: 0.43-0.66) and high LE8 group (OR:0.18, 95% CI: 0.08-0.42) compared to the low LE8 group. Within the same model, the risk of DKD was decreased by 45% and 78% respectively in the high health behaviors score group (OR:0.55, 95% CI: 0.39-0.77) and high health factors score group (OR:0.22, 95% CI: 0.11-0.45). An increase of 10 points in LE8 score (OR:0.71, 95% CI: 0.65-0.78), health behaviors score (OR:0.89, 95% CI: 0.84-0.95), and health factors score (OR:0.75, 95% CI: 0.69-0.82) was associated with a 29%, 11%, and 25% reduction in the risk of DKD, respectively. Furthermore, Figure 3(a–c) exhibited the linear associations between the OR values of DKD and LE8 (*p* for non-linearity: 0.729), health behaviors score (*p* for non-linearity: 0.334), and health factors score (*p* for non-linearity: 0.125), respectively.

**Table 2.** Association of the LE8 and its subscales with DKD.

	Crude model		Model 1		Model 2		Model 3	
	OR (95%CI)	*p* value	OR (95%CI)	*p* value	OR (95%CI)	*p* value	OR (95%CI)	*p* value
LE8 score								
Low	Ref		Ref		Ref		Ref	
Moderate	0.55 (0.45-0.66)	<0.001	0.52 (0.42-0.64)	<0.001	0.52 (0.42-0.64)	<0.001	0.54 (0.43-0.66)	<0.001
High	0.14 (0.06-0.32)	<0.001	0.18 (0.08-0.40)	<0.001	0.18 (0.08-0.41)	<0.001	0.18 (0.08-0.42)	<0.001
Per 10-point increase	0.73 (0.68-0.78)	<0.001	0.71 (0.65-0.77)	<0.001	0.71 (0.65-0.77)	<0.001	0.71 (0.65-0.78)	<0.001
Health behaviors score								
Low	Ref		Ref		Ref		Ref	
Moderate	0.72 (0.58-0.88)	0.002	0.71 (0.56-0.90)	0.005	0.72 (0.57-0.91)	0.006	0.73 (0.58-0.93)	0.012
High	0.53 (0.39-0.71)	<0.001	0.53 (0.38-0.74)	<0.001	0.53 (0.38-0.74)	<0.001	0.55 (0.39-0.77)	<0.001
Per 10-point increase	0.89 (0.84-0.93)	<0.001	0.89 (0.84-0.94)	<0.001	0.89 (0.84-0.94)	<0.001	0.89 (0.84-0.95)	<0.001
Health factors score								
Low	Ref		Ref		Ref		Ref	
Moderate	0.57 (0.48-0.68)	<0.001	0.53 (0.44-0.65)	<0.001	0.54 (0.44-0.65)	<0.001	0.54 (0.44-0.66)	<0.001
High	0.16 (0.08-0.32)	<0.001	0.21 (0.10-0.43)	<0.001	0.21 (0.10-0.44)	<0.001	0.22 (0.11-0.45)	<0.001
Per 10-point increase	0.77 (0.72-0.82)	<0.001	0.75 (0.69-0.81)	<0.001	0.75 (0.69-0.81)	<0.001	0.75 (0.69-0.82)	<0.001

Model 1: adjusted for gender, age, marital status, race, educational level, and family PIR. Model 2: adjusted for model 1 and alcohol consumption status. Model 3: adjusted for gender, age, marital status, race, educational level, family PIR, alcohol consumption status, cardiovascular disease and depression.

OR: odds ratio; CI: confidence interval; LE8: Life’s essential 8; DKD: diabetic kidney disease.

### 3.3. Subgroup and sensitivity analysis

**Figure 4.**
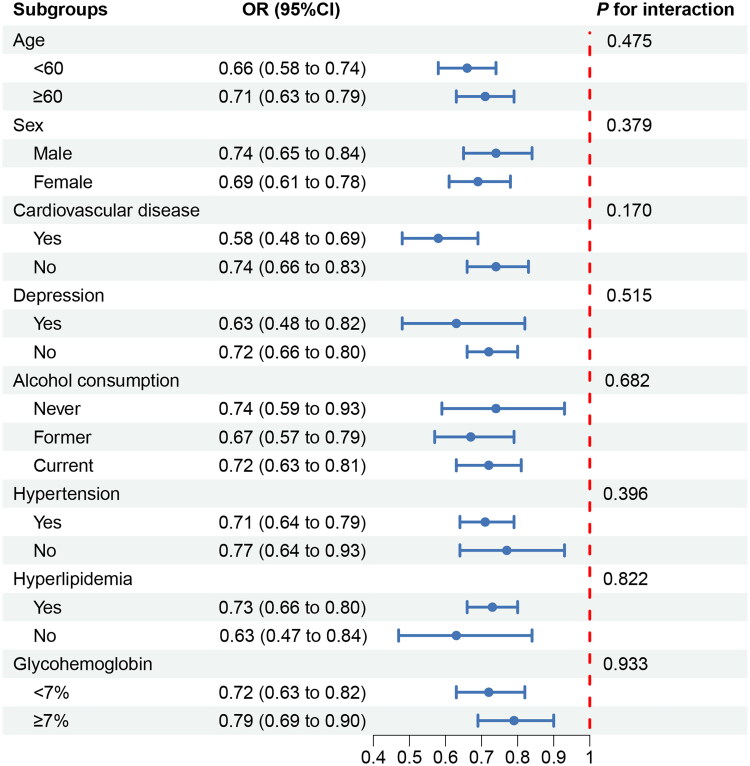
Associations between LE8 and DKD in different subgroups.

The subgroup analysis results are presented in Figure 4. Across subgroups stratified by age, gender, glycohemoglobin level, CVD, hypertension, hyperlipidemia, depression and alcohol use, the LE8 score (per 10-point increase) exhibited a significant negative correlation with the risk of DKD (*p* < 0.05), without notable interactions between the stratifying factors (all *p*-values for interaction tests > 0.05). After excluding participants with CVD and repeating the multivariable logistic regression analysis (Table 3), the findings remained consistent with the previous results after adjusting for all covariates. Specifically, both LE8 score and health factors score demonstrated significant negative associations with the risk of DKD, whereas no significant correlation was observed between health behaviors score and DKD. The results of the multivariate logistic regression, following multiple imputation for missing covariate data, remained consistent (Supplementary Table 4). Overall, the results of the sensitivity analysis were robust.

**Table 3.** Sensitivity analysis.

	Excluding participants with CVD history	
	OR (95% CI)	*P* value
LE8 score		
Low (0–49)	Ref	
Moderate (50–79)	0.61 (0.47-0.80)	<0.001
High (80–100)	0.21 (0.09-0.51)	<0.001
Per 10-point increase	0.74 (0.67-0.83)	<0.001
Health behaviors score		
Low (0–49)	Ref	
Moderate (50–79)	0.79 (0.58-1.07)	0.122
High (80–100)	0.70 (0.48-1.04)	0.075
Per 10-point increase	0.93 (0.87-1.00)	0.044
Health factors score		
Low (0–49)	Ref	
Moderate (50–79)	0.58 (0.45-0.75)	<0.001
High (80–100)	0.19 (0.08-0.46)	<0.001
Per 10-point increase	0.76 (0.68-0.84)	<0.001

OR: odds ratio; CI: confidence interval; CVD, cardiovascular disease; LE8, Life’s essential 8.

## 4. Discussion

To investigate the relationship between LE8 and DKD, we conducted a cross-sectional study involving 3,715 participants, which represents approximately 18.95 million diabetic patients in the United States. The study results indicated that higher LE8 scores were associated with a lower risk of developing DKD. Additionally, we observed a similar trend in the association between health behaviors and health factor scores and DKD. This correlation remained consistent after stratification by age, gender, presence of CVD and depression, and alcohol use status. Even after excluding participants with CVD and multiple imputation for missing covariate data, the significance of this association remained unchanged. Overall, our findings provide preliminary evidence that maintaining a higher level of CVH may be an important way to promote and maintain health in diabetic patients.

To the best of our knowledge, this is the first study leveraging the NHANES to evaluate the relationship between CVH, as assessed by LE8, and DKD. Prior to this, numerous studies have explored the link between CVH and kidney diseases. A prospective study conducted in China revealed that over a 14-year follow-up period, participants with higher LE8 scores exhibited a significantly lower risk of developing DKD compared to those with lower scores [20]. In the United States, a cohort study evaluating the duration of ideal CVH during adulthood and its association with cardiometabolic outcomes and mortality showed that individuals with moderate or ideal CVH in midlife had reduced risks of CKD, diabetes, and all-cause mortality compared to those with poorer CVH [21]. Research based on the NHANES has also found a negative correlation between CVH and the prevalence of CKD, particularly pronounced among individuals aged 65 and older [22–24]. Furthermore, among individuals already diagnosed with CKD, maintaining a higher LE8 level was associated with a reduced risk of all-cause and cause-specific mortality. Given that diabetics constitute one of the most vulnerable populations to kidney damage, the presence of DKD complicates diabetes management and elevates the risks of CVD and death. Our study, utilizing the NHANES and defining CVH through LE8, is the first to explore the association between CVH and DKD risk specifically in the diabetic population. While our study population differs in terms of ethnicity and specific patient groups from those in previous studies, our findings suggest that a higher LE8 level is similarly associated with a reduced risk of DKD.

Our research further illuminated the linear relationship between LE8 and its constituent scales with DKD, reinforcing the evidence underpinning their association. Our findings revealed that the ORs for the correlation between LE8, health behavior scores, and health factor scores with DKD exhibited a steady decline as the scores increased, with saturation effects observed in both LE8 and health factors. This suggests that optimizing health factors can indeed effectively mitigate the risk of DKD. However, this protective effect exhibits an upper limit, implying that once health factors are optimized to a certain level, further improvements yield diminishing returns in reducing DKD risk. Consequently, future research could delve deeper into the specific mechanisms underlying this saturation effect and explore multifaceted interventions to maximize the reduction of DKD risk, thereby informing the formulation of public health policies and clinical interventions, and averting excessive resource allocation and waste. Conversely, the absence of such a saturation effect in the relationship between health behavior factors and DKD underscores the potential value of stricter health behavior standards. Although our subgroup analysis stratified by age, gender, glycohemoglobin level, CVD, hypertension, hyperlipidemia, depression, and alcohol use did not reveal significant interactions, further validating the robustness of our results, this finding underscores the importance of focusing on individual variations. It highlights the need to identify subpopulations that remain at elevated risk of DKD, despite adopting high levels of healthy behaviors or possessing favorable health factors, thereby facilitating the adoption of more precise and personalized preventive strategies.

The precise mechanisms underlying diabetic vascular complications remain incompletely understood, with hyperglycemia-induced endothelial dysfunction widely recognized as a common initiating factor for both diabetic cardiovascular and renal diseases [25]. Endothelial dysfunction is defined as the loss of endothelial vasodilatory, anticoagulant, and anti-inflammatory properties, primarily mediated by reduced nitric oxide (NO) availability, leading to arterial wall vasoconstriction, thrombosis, and inflammation [26, 27]. In diabetic CVD, which is predominantly manifested as atherosclerosis, endothelial dysfunction of the arterial intima is characterized by decreased NO bioavailability, along with increased production or action of endothelial-derived vasoconstrictors. In diabetic nephropathy, endothelial dysfunction manifests as reduced NO release, enhanced oxidative stress, and increased production of inflammatory factors. As NO bioavailability decreases in large vessels, the expression of endothelial nitric oxide synthase (eNOS) and NO production in microvessels also diminish. Reduced eNOS expression has been observed in cardiac tissue [28–32] and glomeruli [33–35] of diabetic animal models, and increasing NO levels has been shown to improve cardiomyopathy, reduce proteinuria, and delay renal damage [36, 37]. Endothelial dysfunction in diabetes is intricately linked to oxidative stress [38], low-grade inflammation [39, 40], abnormal adipokine levels, and Endothelial-to-mesenchymal transition. Elevated low-density lipoprotein (LDL)-cholesterol promotes endothelial dysfunction by increasing reactive oxygen species production and eNOS uncoupling [41]. In insulin resistance, reduced NO availability contributes to impaired endothelial function, while hyperglycemia augments inflammation and vasoconstrictor production, further damaging endothelial vessels [42, 43]. Smoking exacerbates inflammation, thrombosis, and LDL-cholesterol oxidation, thus intensifying oxidative stress [44]. The combination of exercise and dietary control can improve glycemic and lipid metabolism in obese adolescents by modulating microRNA-126, leading to enhanced microvascular endothelial function [45]. Short sleep duration disrupts autonomic nerve balance and the circadian rhythm of peripheral vascular clock components, contributing to endothelial dysfunction [46]. Consequently, both health factors and behavioral factors within LE8 have the potential to influence endothelial function, leading to diabetic macrovascular and microvascular complications. This underscores the potential of LE8 as an important tool for assessing the risk of DKD.

DKD imposes significant economic and daily life burdens on patients. Consequently, management strategies for DKD should prioritize early diagnosis, prevention, and the delay of progressive renal dysfunction. Our study findings further indicate that only 3% of participants achieved high CVH, while 32.2% fell into the low CVH category, highlighting the minimal genetic influence on CVH [47] and the dominant role of behavioral and environmental factors. The AHA also advocates for enhancing public awareness of CVH, reinforcing routine and standardized assessments of diet, physical activity, nicotine exposure, and sleep health in both clinical and population settings [11]. Healthcare systems, individuals, and families can contribute to the pragmatic collection of CVH data, leveraging public health platforms to facilitate long-term monitoring and professional evaluations of CVH. Progress tracking through online websites or applications is encouraged. These health technology platforms can aggregate CVH data for population health surveillance and risk prediction, or leverage incentives to motivate behavioral changes across diverse populations, thereby improving CVH. Our research underscores that for diabetics, adhering to comprehensive management and treatment guidelines such as LE8 may reduce the risk of DKD and enhance overall health. Clinicians and patients should collaborate to prioritize and intensify strategies that enhance success rates and sustainably maintain high levels of overall CVH. Given adherence challenges, patients may be advised to commence with focusing on a single component, cultivating long-term commitment for meaningful improvements.

## 5. Strengths and limitations

The adoption of LE8, a comprehensive quantitative indicator, as a health assessment tool in this study offers several advantages. Primarily, it integrates multiple health factors, circumventing the limitations of traditional research that focuses solely on singular health aspects. This holistic assessment approach better mirrors real-world health status, enhancing the scientific rigor and credibility of the findings. Leveraging the NHANES database, the study employed a sophisticated, multi-stage probability sampling design, bolstering the representativeness and generalizability of the results through weighting adjustments. The preliminary findings reveal a correlation between LE8 and DKD, suggesting the potential of LE8 as a risk stratification tool for diabetes, thereby offering novel insights for developing personalized prevention strategies. However, several limitations must be acknowledged. Firstly, the cross-sectional design of the study prohibits the establishment of a causal relationship between LE8 and DKD, as well as the observation of the association between sustained high LE8 scores and DKD outcomes. Consequently, longitudinal studies are warranted to explore the specific mechanisms underlying the influence of LE8 on DKD. Secondly, LE8 data relies heavily on self-reported dietary and lifestyle information from participants, which may introduce recall and selection biases, compromising the credibility of the information. Nevertheless, given the accessibility of current data and NHANES’ status as a recommended source for tracking LE8 by the AHA, the research outcomes based on this methodology retain a degree of value. Furthermore, despite NHANES’ adoption of stratified, multi-stage sampling methods, potential biases such as non-response bias and self-selection bias may persist, potentially hindering the complete reflection of the true population picture. Although we have addressed some known biases by applying weighting methods recommended in the NHANES guidelines, caution is still advised in interpreting the results. Lastly, as the study participants were exclusively from the United States, the applicability of the findings to diverse racial groups necessitates additional validation in more multicultural settings.

## 6. Conclusions

This study preliminarily unveils a correlation between higher LE8 and a lower risk of developing DKD among diabetic patients. This finding suggests that LE8 can aid in identifying patients with a higher risk of DKD, while also offering novel insights into the development of personalized dietary planning and lifestyle guidance frameworks for diabetic patients. It underscores the potential value of LE8 in adjusting public health strategies. However, the current research conclusions necessitate further validation and refinement through large-scale, rigorously designed prospective studies to explore the long-term causal relationship between LE8 and DKD, as well as the potential differential effects that may exist across different populations and individuals.

## Supplementary Material

Supplemental Material

Supplemental Material

Supplemental Material

Supplemental Material

Supplemental Material
