# Supplementary material for: Association between life’s essential 8 and diabetic kidney disease: a population-based study
Source: Ren Fail. 2025 Mar 10;47(1):2454286. doi: 10.1080/0886022X.2025.2454286 (PMC11894740; doi:10.1080/0886022X.2025.2454286)
Supplement: Supplemental Material [file IRNF_A_2454286_SM7472.docx]

**Supplementary Table 1** Healthy Eating Index-2015 Components & Scoring Standards ^1^

| Component | Maximum points | Standard for maximum score | Standard for minimum score of zero |
| --- | --- | --- | --- |
| Adequacy |  |  |  |
| Total fruits^2^ | 5 | ≥ 0.8 cup equivalents per 1,000 kcal | No fruits |
| Whole fruits^3^ | 5 | ≥ 0.4 cup equivalents per 1,000 kcal | No whole fruits |
| Total vegetables^4^ | 5 | ≥ 1.1 cup equivalents per 1,000 kcal | No vegetables |
| Greens and beans^4^ | 5 | ≥ 0.2 cup equivalents per 1,000 kcal | No dark green vegetables or legumes |
| Whole grains | 10 | ≥ 1.5 oz equivalents per 1,000 kcal | No whole grains |
| Dairy^5^ | 10 | ≥ 1.3 cup equivalents per 1,000 kcal | No dairy |
| Total protein foods^6^ | 5 | ≥ 2.5 oz equivalents per 1,000 kcal | No protein foods |
| Seafood and plant proteins^6,7^ | 5 | ≥ 0.8 oz equivalents per 1,000 kcal | No seafood or plant proteins |
| Fatty acids^8^ | 10 | (PUFAs + MUFAs)/SFAs ≥ 2.5 | (PUFAs + MUFAs)/SFAs ≤ 1.2 |
| Moderation |  |  |  |
| Refined grains | 10 | ≤ 1.8 oz equivalents per 1,000 kcal | ≥ 4.3 oz equivalents per 1,000 kcal |
| Sodium | 10 | ≤ 1.1 gram per 1,000 kcal | ≥ 2.0 grams per 1,000 kcal |
| Added sugars | 10 | ≤ 6.5% of energy | ≥ 26% of energy |
| Saturated fats | 10 | ≤ 8% of energy | ≥ 16% of energy |

(1) Intakes between the minimum and maximum standards are scored proportionately.

(2) Includes 100% fruit juice.

(3) Includes all forms except juice.

(4) Includes legumes (beans and peas).

(5) Includes all milk products, such as fluid milk, yogurt, and cheese, and fortified soy beverages.

(6) Includes legumes (beans and peas).

(7) Includes seafood, nuts, seeds, soy products (other than beverages), and legumes (beans and peas).

(8) Ratio of poly- and monounsaturated fatty acids (PUFAs and MUFAs) to saturated fatty acids (SFAs).

Adequacy components represent the food groups, subgroups, and dietary elements that are encouraged. For these components, higher scores reflect higher intakes, because higher intakes are desirable.

Moderation components represent the food groups and dietary elements for which there are recommended limits to consumption. For moderation components, higher scores reflect lower intakes, because lower intakes are more desirable.

**Reference**

1. Krebs-Smith SM, Pannucci TE, Subar AF, et al. Update of the Healthy Eating Index: HEI-2015. J Acad Nutr Diet. Sep 2018;118(9):1591-1602.
