# Supplementary material for: Association between life’s essential 8 and diabetic kidney disease: a population-based study
Source: Ren Fail. 2025 Mar 10;47(1):2454286. doi: 10.1080/0886022X.2025.2454286 (PMC11894740; doi:10.1080/0886022X.2025.2454286)
Supplement: Supplemental Material [file IRNF_A_2454286_SM7470.docx]

**Supplementary Table 3** The collinearity assessment outcomes

| variables | GVIF | df | GVIF^(1/(2*df)) |
| --- | --- | --- | --- |
| Age | 1.389 | 1 | 1.178 |
| Sex | 1.177 | 1 | 1.085 |
| Race and ethnicity | 1.285 | 3 | 1.043 |
| Educational level | 1.343 | 2 | 1.076 |
| Marriage status | 1.257 | 2 | 1.059 |
| Family PIR | 1.472 | 2 | 1.101 |
| Cardiovascular disease | 1.175 | 1 | 1.084 |
| Depression | 1.101 | 1 | 1.049 |
| Alcohol use | 1.412 | 4 | 1.044 |

PIR: poverty income ratio; VIF: variance inflation factor.
