# Supplementary material for: Association between life’s essential 8 and diabetic kidney disease: a population-based study
Source: Ren Fail. 2025 Mar 10;47(1):2454286. doi: 10.1080/0886022X.2025.2454286 (PMC11894740; doi:10.1080/0886022X.2025.2454286)
Supplement: Supplemental Material [file IRNF_A_2454286_SM7469.docx]

**Supplementary Table 4** Sensitivity analysis

| Variable | Model 3 | | | | |
| --- | --- | --- | --- | --- | --- |
|  | Imput database 1 | Imput database 2 | Imput database 3 | Imput database 4 | Imput database 5 |
|  | OR (95%CI) | OR (95%CI) | OR (95%CI) | OR (95%CI) | OR (95%CI) |
| LE8 score |  |  |  |  |  |
| Low | Ref | Ref | Ref | Ref | Ref |
| Moderate | 0.53 (0.44, 0.63) | 0.52 (0.44, 0.63) | 0.52 (0.44, 0.63) | 0.52 (0.43, 0.63) | 0.52 (0.43, 0.63) |
| High | 0.21 (0.10, 0.44) | 0.20 (0.10, 0.43) | 0.20 (0.10, 0.43) | 0.20 (0.10, 0.43) | 0.20 (0.09, 0.43) |
| Per 10-point increase | 0.72 (0.66, 0.78) | 0.72 (0.66, 0.77) | 0.72 (0.66, 0.78) | 0.71 (0.66, 0.77) | 0.71 (0.66, 0.77) |
| Health behaviors score |  |  |  |  |  |
| Low | Ref | Ref | Ref | Ref | Ref |
| Moderate | 0.73 (0.58, 0.91) | 0.72 (0.58, 0.91) | 0.72 (0.58, 0.91) | 0.72 (0.58, 0.91) | 0.72 (0.57, 0.90) |
| High | 0.54 (0.39, 0.74) | 0.53 (0.39, 0.73) | 0.54 (0.39, 0.74) | 0.53 (0.38, 0.73) | 0.53 (0.39, 0.73) |
| Per 10-point increase | 0.89 (0.85, 0.94) | 0.89 (0.85, 0.94) | 0.89 (0.85, 0.94) | 0.89 (0.84, 0.94) | 0.89 (0.84, 0.94) |
| Health factors score |  |  |  |  |  |
| Low | Ref | Ref | Ref | Ref | Ref |
| Moderate | 0.56 (0.47, 0.67) | 0.56 (0.47, 0.67) | 0.56 (0.46, 0.67) | 0.56 (0.47, 0.67) | 0.56 (0.46, 0.67) |
| High | 0.26 (0.13, 0.52) | 0.26 (0.13, 0.51) | 0.27 (0.14, 0.53) | 0.26 (0.13, 0.52) | 0.26 (0.13, 0.51) |
| Per 10-point increase | 0.76 (0.71, 0.82) | 0.76 (0.71, 0.82) | 0.76 (0.70, 0.82) | 0.76 (0.71, 0.82) | 0.76 (0.70, 0.82) |

Model 3: adjusted for gender, age, marital status, race, educational level, and family PIR, alcohol consumption status, cardiovascular disease and depression.

OR: odds ratio; CI: confidence interval; LE8: Life's essential 8; DKD: diabetic kidney disease.
