# Supplementary material for: Association between life’s essential 8 and diabetic kidney disease: a population-based study
Source: Ren Fail. 2025 Mar 10;47(1):2454286. doi: 10.1080/0886022X.2025.2454286 (PMC11894740; doi:10.1080/0886022X.2025.2454286)
Supplement: Supplemental Material [file IRNF_A_2454286_SM7468.docx]

**Supplementary Table 5** Baseline Characteristics of the Study Population

| Characteristics | Overall | Non-DKD | DKD | *p* |
| --- | --- | --- | --- | --- |
| Participant number (weighted) | n= 18952666 | n= 12055286 | n= 6897380 |  |
| Age, years | 59.72 (13.35) | 56.94 (12.92) | 64.59 (12.70) | <0.0001 |
| Sex, % |  |  |  | 0.975 |
| Male | 50.45 | 50.48 | 50.40 |  |
| Female | 49.55 | 49.52 | 49.60 |  |
| Race, % |  |  |  | 0.0205 |
| Non-Hispanic White | 66.61 | 65.69 | 68.23 |  |
| Non-Hispanic Black | 13.35 | 12.90 | 14.13 |  |
| Mexican American | 8.14 | 8.15 | 8.13 |  |
| Other | 11.90 | 13.27 | 9.51 |  |
| Education level, % |  |  |  | 0.0129 |
| Less than high school | 20.68 | 19.16 | 23.34 |  |
| High school diploma | 26.62 | 25.23 | 29.06 |  |
| More than high school | 52.69 | 55.61 | 47.60 |  |
| Marry status, % |  |  |  | <0.0001 |
| Divorced/separated/widowed | 27.00 | 23.45 | 33.21 |  |
| Married/living with a partner | 65.19 | 68.16 | 60.00 |  |
| Never married | 7.81 | 8.39 | 6.78 |  |
| Family PIR, % |  |  |  | <0.0001 |
| <1.3 | 23.48 | 20.94 | 27.91 |  |
| 1.3-3.5 | 39.71 | 37.96 | 42.77 |  |
| ≥3.5 | 36.81 | 41.10 | 29.32 |  |
| Cardiovascular disease, % |  |  |  | <0.0001 |
| No | 74.99 | 80.89 | 64.66 |  |
| Yes | 25.01 | 19.11 | 35.34 |  |
| Depression, % |  |  |  | 0.9029 |
| No | 89.08 | 89.14 | 88.98 |  |
| Yes | 10.92 | 10.86 | 11.02 |  |
| Alcohol consumption, % |  |  |  | 0.0002 |
| Never | 13.98 | 13.14 | 15.46 |  |
| Former | 23.33 | 20.65 | 28.03 |  |
| Current | 62.69 | 66.21 | 56.51 |  |
| AHA LE8 scores |  |  |  |  |
| Total LE8 score | 55.67 (13.20) | 57.62 (13.00) | 52.25 (12.87) | <0.0001 |
| Health behaviors score | 63.23 (19.04) | 64.83 (18.87) | 60.41 (19.00) | <0.0001 |
| Diet score | 50 (25,80) | 50 (25,80) | 50 (25,80) | 0.9562 |
| Physical activity score | 80 (0,100) | 93.80 (0,100) | 40 (0,100) | <0.0001 |
| Smoke score | 75 (75,100) | 80 (75,100) | 75 (75,100) | 0.5601 |
| Sleep score | 80.85 (25.79) | 82.30 (24.84) | 78.31 (27.20) | 0.0045 |
| Health factors score | 48.11 (15.89) | 50.41 (15.85) | 44.09 (15.15) | <0.0001 |
| BMI score | 30 (15,70) | 30 (15,70) | 30 (15,70) | 0.0505 |
| Blood lipids score | 60 (40,80) | 60 (40,80) | 60 (40,80) | 0.3706 |
| Blood glucose score | 40 (30,40) | 40 (30,40) | 40 (30,40) | <0.0001 |
| Blood pressure score | 50 (30,80) | 55 (30,80) | 30 (5,75) | <0.0001 |
| ACR, mg/g |  |  |  | <0.0001 |
| eGFR, mL/min/1.73 m^2^ | 82.13 (24.25) | 90.82 (17.06) | 66.88 (27.28) | <0.0001 |

PIR: poverty income ratio; AHA: American Heart Association; LE8: life's essential 8; BMI: body mass index; ACR: urinary albumin-to-creatinine ratio; eGFR: estimated glomerular filtration rate; DKD: diabetic kidney disease.
